# The Expanding Field of Secondary Antibody Deficiency: Causes, Diagnosis, and Management

**DOI:** 10.3389/fimmu.2019.00033

**Published:** 2019-02-08

**Authors:** Smita Y. Patel, Javier Carbone, Stephen Jolles

**Affiliations:** ^1^Clinical Immunology Department, Oxford University Hospitals NHS Foundation Trust, Oxford, United Kingdom; ^2^Clinical Immunology Department, Hospital General Universitario Gregorio Marañon, Madrid, Spain; ^3^Immunodeficiency Centre for Wales, University Hospital of Wales, Cardiff, United Kingdom

**Keywords:** secondary antibody deficiency, chronic lymphocytic leukemia, lymphoma, multiple myeloma, solid organ transplant, immunoglobulin replacement (IgRT)

## Abstract

Antibody deficiency or hypogammaglobulinemia can have primary or secondary etiologies. Primary antibody deficiency (PAD) is the result of intrinsic genetic defects, whereas secondary antibody deficiency may arise as a consequence of underlying conditions or medication use. On a global level, malnutrition, HIV, and malaria are major causes of secondary immunodeficiency. In this review we consider secondary antibody deficiency, for which common causes include hematological malignancies, such as chronic lymphocytic leukemia or multiple myeloma, and their treatment, protein-losing states, and side effects of a number of immunosuppressive agents and procedures involved in solid organ transplantation. Secondary antibody deficiency is not only much more common than PAD, but is also being increasingly recognized with the wider and more prolonged use of a growing list of agents targeting B cells. SAD may thus present to a broad range of specialties and is associated with an increased risk of infection. Early diagnosis and intervention is key to avoiding morbidity and mortality. Optimizing treatment requires careful clinical and laboratory assessment and may involve close monitoring of risk parameters, vaccination, antibiotic strategies, and in some patients, immunoglobulin replacement therapy (IgRT). This review discusses the rapidly evolving list of underlying causes of secondary antibody deficiency, specifically focusing on therapies targeting B cells, alongside recent advances in screening, biomarkers of risk for the development of secondary antibody deficiency, diagnosis, monitoring, and management.

## Introduction

**Figure 1:**
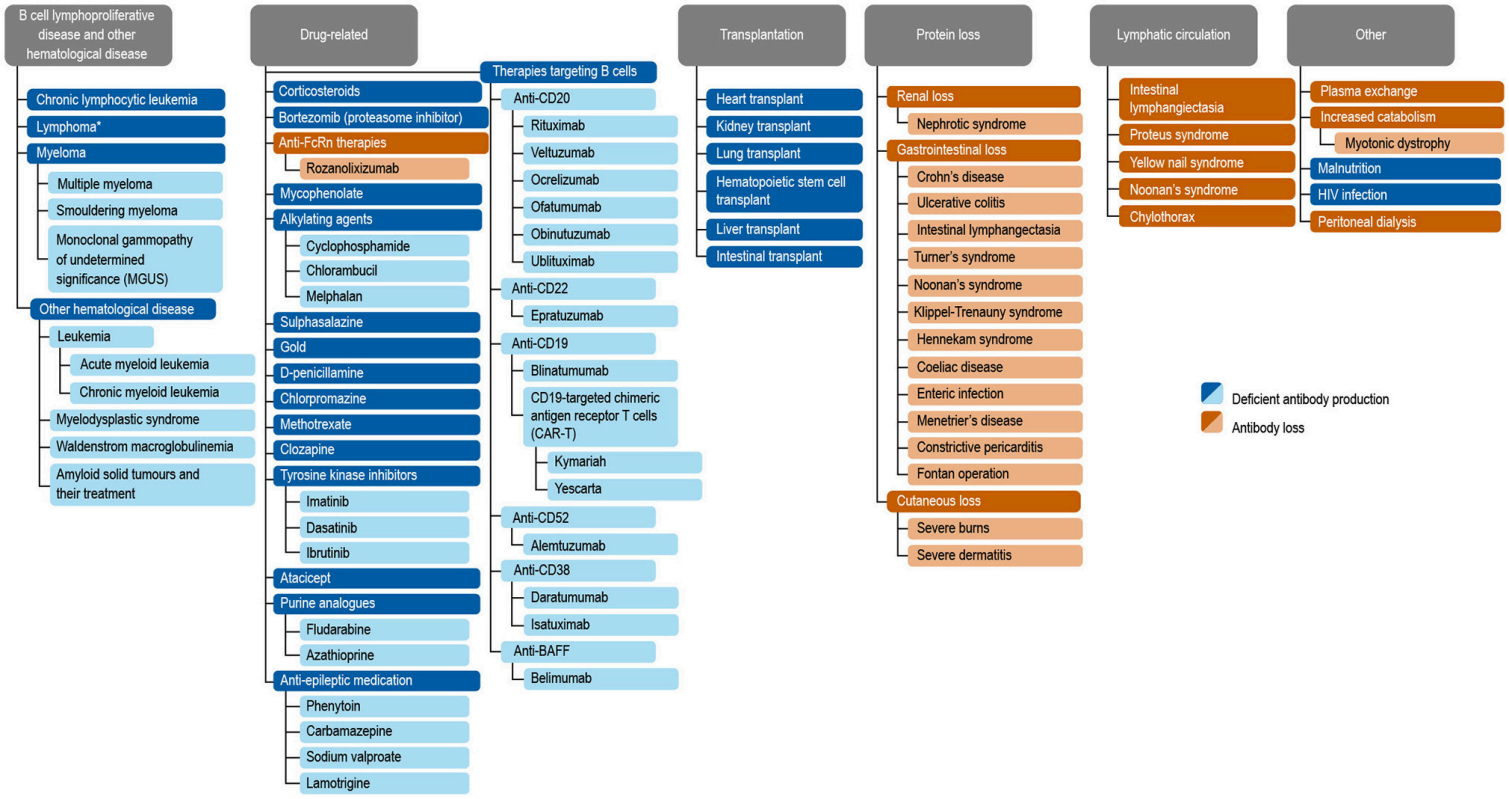
Common causes of secondary antibody deficiency (26), (5), (251), (255), (256), (242), (6), (144), (7), (165), (70), (242), (18), (168), (244), (134), (141), (135), (139), (133), (155), (245), (147), (138), (38), (162), (124), (163), (246), (174), (233), (253), (257), (247), (252), (249), (250), (10), (254). Reproduced with the permission of the copyright holder John Wiley & Sons Inc (5). ^*^Including non-Hodgkin's lymphoma, Hodgkin's Lymphoma, diffuse large B cell lymphoma, follicular lymphoma, mantle cell lymphoma, marginal zone lymphoma, and Burkitt's lymphoma (5).

Antibody deficiencies, a subset of immunodeficiencies, are classified as primary or secondary in etiology. Primary antibody deficiency (PAD) refers to a heterogeneous group of genetic disorders characterized by an intrinsic impairment in antibody production or function (1, 2). The prevalence of PAD has been estimated to be around 1 in 2,000 children, 1 in 1,200 individuals of any age, and 1 in 600 households in the United States (~150,000–360,000 patients) (3). Secondary immunodeficiencies (SID) on a worldwide scale occur as a consequence of an extrinsic influences, such as malnutrition, human immunodeficiency virus (HIV) infection, malaria, neutropenia, or as a side effect of certain medications (4). Secondary antibody deficiency, a type of SID, is often multifactorial in etiology, related to both the underlying condition and its treatment, including a growing range of treatments targeting B cells. Secondary antibody deficiency occurs across a broad wide disease spectrum, and is therefore of importance to clinicians in both primary and secondary care. Secondary antibody deficiencies are can be estimated to be 30-fold more common than PADs, but unlike PADs are sometimes reversible if the underlying cause is resolved (4). There are several types of secondary antibody deficiency, the most common being disease-related secondary antibody deficiency, caused by hematologic malignancies such as chronic lymphocytic leukemia (CLL), lymphoma, and multiple myeloma (MM). Other types of secondary antibody deficiency include iatrogenic secondary antibody deficiency as a side effect of specific therapies designed to target B cells directly as well as non-B cell specific therapies or processes which nonetheless impact on B cells or antibodies including conventional immunosuppressive agents (e.g., cyclophosphamide, methotrexate, mycophenolate mofetil) steroids, radiation therapy, solid organ transplantation (SOT), and secondary antibody deficiency related to protein-losing states due to renal, gastrointestinal, or cutaneous loss (Figure 1) (2, 4–6). Patients with secondary antibody deficiency due to renal or gastrointestinal loss of IgG often have retained specific antibody production and hence may have a lower infection risk when compared to a failure to produce antibodies (2). The removal of antibodies by plasma exchange or blockade of the neonatal Fc receptor (FcRn) is also likely to confer a lower risk of infection than deficiencies in antibody production (7–10).

The spectrum of clinical impact of secondary antibody deficiency may range from a rather limited infection susceptibility to a more significant burden characterized by predominantly sinopulmonary recurrent, chronic, systemic, or complicated infections or even including opportunistic infections, such as cytomegalovirus (CMV) in transplantation (5). Although secondary antibody deficiency is a recognized phenomenon, there are only a limited number of studies addressing the incidence and clinical importance of this disorder (11) and treatment outcomes have not been fully explored.

The prevalence of secondary antibody deficiency, which is estimated to be 30 times more common than PAD (12–15), is increasing for a number of reasons. Growth in new therapies for autoimmune, inflammatory, and malignant disease, many targeting B cells, is rapid. It is being increasingly appreciated that the use of such therapies, such as rituximab, alongside high-dose, and long-term steroid treatment, either in combination with other treatments, sequentially over time or as maintenance therapies may contribute to the increased development of iatrogenic secondary antibody deficiency (5, 6). Studies report an increased risk of infection with B cell-targeting drugs such as rituximab or ibrutinib, and with long-term steroid treatment (16, 17). It is becoming increasingly important to address the unmet needs in this growing patient population, including risk factors for the development of secondary antibody deficiency, improved screening, monitoring, and treatment strategies.

The clinical management of secondary antibody deficiency involves a range of potential interventions based on a careful clinical and laboratory assessment of risk and may include patient education, prompt access to emergency antibiotics, prophylactic antibiotic treatment, vaccination, and reducing immunosuppression or treating the underlying cause in the small proportion where this is possible. A trial of immunoglobulin replacement therapy (IgRT) may be warranted for selected patients with low immunoglobulin G (IgG; <4 g/L) who continue to suffer recurrent infections despite prophylactic antibiotics and who fail to respond to vaccination. Available studies report IgRT as an effective treatment for reducing the rate of serious infections in patients with CLL or MM (17–19). Recent market research of secondary specialty pharmacy data from the United States, France, the United Kingdom, Germany, Spain, Australia and Canada reported that the major secondary antibody deficiency indications leading to IgRT usage were CLL and MM, which represent 39.2–54.9% of all patients with secondary antibody deficiency receiving IgRT (20).

**Figure 2:**
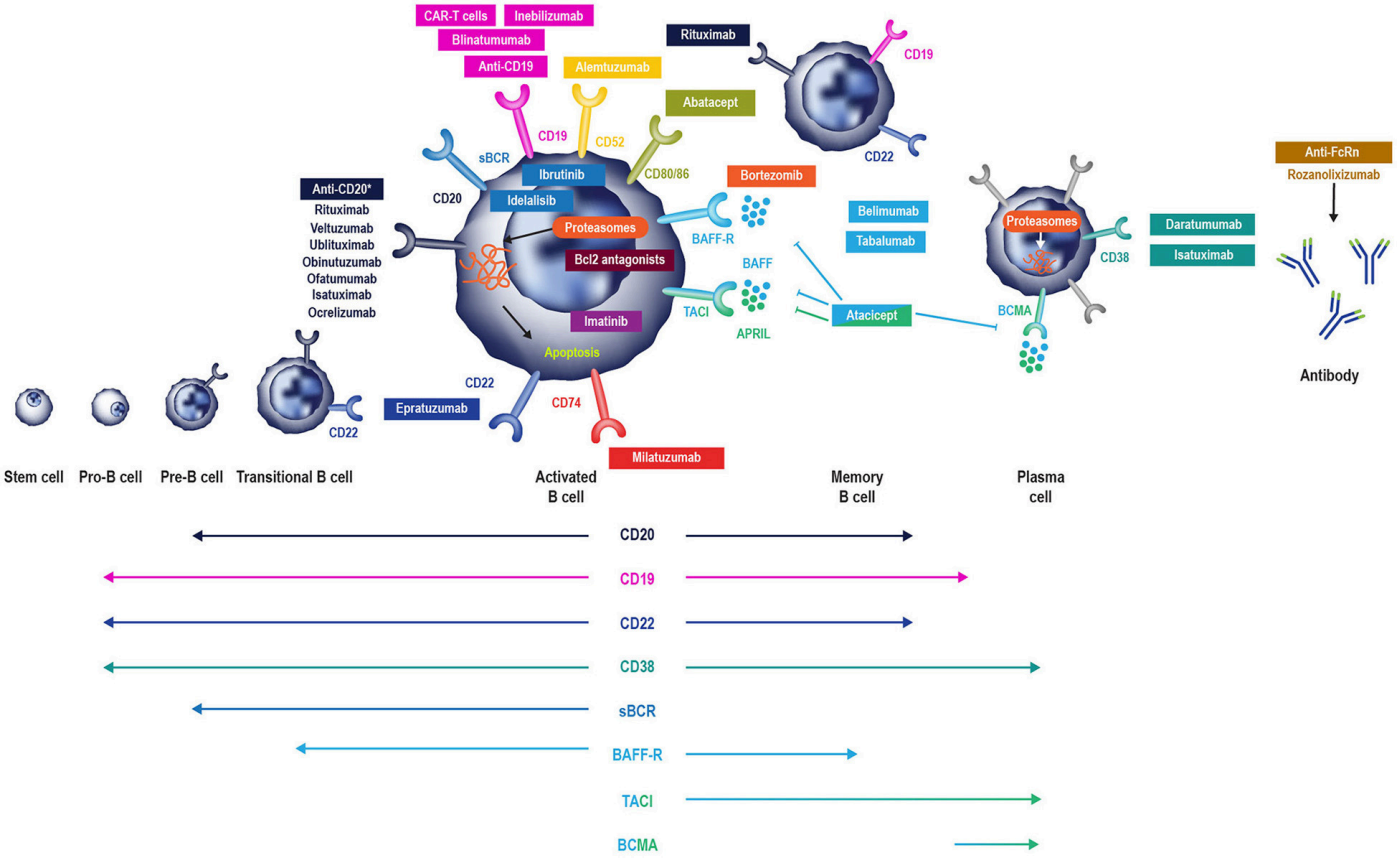
B cell-specific chemotherapeutic causes of secondary antibody deficiency. Reproduced with the permission of the copyright holder The Royal College of Physicians (2). ^*^Anti-CD20 compounds conjugated to other drugs are also in development. APRIL, a proliferation inducing ligand; BAFF(-R), B-cell activating factor (receptor); Bcl2, B cell lymphoma 2; BCMA, B-cell maturation antigen; (s)BCR, (surface) B cell receptor; TACI, transmembrane activator, and calcium modulator.

In this review, we discuss the causes, diagnosis, and management of secondary antibody deficiency, specifically focusing on new developments in agents targeting B cells (Figures 1, 2).

## Causes of Secondary Antibody Deficiency

### CLL, MM, and Lymphoma

Hematological malignancies such as CLL, MM, and lymphoma are commonly associated with hypogammaglobulinemia. CLL is one of the most common leukemias, with an annual incidence of 4.7 in 100,000 (15). Infection frequency in CLL correlates positively with hypogammaglobulinemia, which is present in up to 85% of these patients and contributes substantially to morbidity and mortality, with infection-related deaths in 25–50% of patients (13). The prevalence of hypogammaglobulinemia in CLL is more pronounced with prolonged disease duration and advanced-stage disease and correlates with patient age and comorbidities (13).

Multiple myeloma has an annual incidence of 6.6 cases per 100,000 persons in the United States (14). Secondary antibody deficiency is also common in MM, occurring in 45–83% of patients with smoldering MM at some point during the disease course (21). Infections in such patients are predominantly caused by encapsulated bacteria, such as certain strains of *Haemophilus influenzae*, however overall susceptibility is wide and includes *Clostridium difficile* and *Escherichia coli, Staphylococcus aureus*, and fungal and viral infections, such as *Varicella Zoster* virus (VZV) (6, 22). One study of more than 3,000 patients with MM demonstrated that infection was responsible for 45% of deaths within 6 months of diagnosis. Respiratory tract infections (RTIs) are noted most frequently, with pneumonia, septicemia, and urinary tract infections (UTIs) also occurring commonly in this patient population (6, 23). The hazard ratios of developing pneumonia, septicemia, or meningitis have been shown to be 7.7-, 15.6-, and 16.6-fold, respectively, in patients with MM, compared with population-based age-matched controls (23).

The mechanisms of antibody deficiency and hence infection susceptibility in CLL are multifactorial. Defective function of the non-clonal CD5-negative B cells and direct suppression of CD95^+^ bone marrow plasma cells through CD95L/CD95 interactions between plasma cells and CLL-B cells are postulated to cause a B cell defect (13). Regulatory abnormalities in T cells (e.g., decreased helper T cell or increased T suppressor cell activity) (24) and dysfunctional dendritic cells or natural killer cells may also contribute to the infection burden associated with hypogammaglobulinemia in CLL and MM (2, 6, 13). There is also evidence that CLL-B cells replace normal B cells (25), thereby inhibiting the function of non-malignant B cells by subverting T cell help in the pseudofollicle (26), and may also directly suppress IgG production by bone marrow plasma cells (27). Additional B cell–independent risk factors, such as neutropenia, and significant renal dysfunction can be both disease related and a consequence of treatment. Furthermore, renal disease can act as a cofactor in increasing infection burden not only in CLL and MM but in other settings where there is significant renal impairment (28, 29).

### Therapeutic Agents That Can Cause Secondary Antibody Deficiency

Although CLL and MM can themselves result in secondary antibody deficiency, there is also an additional risk of iatrogenic secondary antibody deficiency posed by the therapies used to treat these, and other conditions (Table 1). Therapies for CLL and MM often suppress immune function, increasing the likelihood of clinically significant infection primarily depending on the actions of the drug, its dose, the duration of treatment, and the stage of CLL (123). According to the market research survey mentioned above, iatrogenic secondary antibody deficiency accounted for 12.8–22.1% of all secondary antibody deficiency cases worldwide (20).

**Table 1:** Reported outcomes of therapeutic agents with the potential to cause iatrogenic secondary antibody deficiency.

**Drug**	**Total number of patients in cited studies**	**Secondary antibody deficiency**	**Infection incidence**	**Vaccine responses**	**B cell subsets**	**References**
Atacicept	697	Decreased IgA, IgG	Increased infection incidence	No effect on tetanus or diphtheria vaccine immunization status	Transiently increased mature and total B cells, followed by decreased mature and total B cells. Transiently increased memory and naïve B cells	(30–37)
Belimumab	4552	Rare minor hypogammaglobulinemia	No change in infection incidence	No effect on pneumococcal, tetanus or influenza vaccine immunization status	Decreased total, transitional, naïve and activated B cells. Possibly transiently increased memory B cells	(38–52)
Bendamustine	396	Hypogammaglobulinemia (IgG)	Increased infection incidence	–	–	(53–55)
Blinatumomab	332	Hypogammaglobulinemia (predominantly IgA)	Possibly increased serious infection incidence	–	Decreased total and peripheral blood cells	(56–61)
Bortezomib	741	Decreased IgG, IgA, and IgM (but not hypogammaglobulinemia)	Increased incidence of HZ infections and VZV reactivation	No effect on measles, mumps or tetanus vaccine immunization status	No effect on B cells	(62–64)
Chlorambucil	24	Decreased IgM (but not hypogammaglobulinemia)	No change in infection incidence	–	–	(65, 66)
Cladribine	205	No effect on immunoglobulins	Increased infection incidence	–	Decreased total B cells	(67–69)
Clozapine	119	Hypogammaglobulinemia (IgG, IgA, and IgM)	–	–	–	(70–72)
Corticosteroids	274	Hypogammaglobulinemia	Increased infection incidence	No effect on influenza vaccine immunization status	Decreased naïve and transitional B cells. No effect on memory B cells	(73–75)
Daratumumab	319	–	Increased infection incidence	–	No effect on B cells	(76, 77)
Epratuzumab	1468	Decreased IgM (no effect/possibly increased IgA or IgG)	–	–	Decreased total B cells	(78–84)
Fludarabine	27	Hypogammaglobulinemia	Increased infection incidence	–	–	(85)
Ibrutinib	894	Hypogammaglobulinemia (IgG, IgA, and IgM)	Increased infection incidence	No effect on influenza vaccine immunization status	–	(16, 86–91)
Idelalisib	267	No effect on immunoglobulins	Increased infection incidence	–	–	(92–95)
Inebilizumab	51	Decreased IgG, IgE, IgG, and IgM (but not hypogammaglobulinemia)	Increased infection incidence	–	Decreased total B cells	(96, 97)
Mycophenolate mofetil	669	Decreased IgG and IgM	Increased CMV and bacterial infection incidence	Suppressed response to influenza vaccine	Decreased plasmablasts	(98–102)
Ocrelizumab	2830	Hypogammaglobulinemia reported (mostly IgM)	Increased infection incidence	No effect on mumps, rubella, varicella, or pneumococcal immunization status	Decreased total and peripheral B cells	(103–110)
Rituximab	500	Hypogammaglobulinemia (IgA, IgG, IgM)	Increased infection incidence	Suppressed response to pneumococcal and *haemophilus influenzae* immunization status. No effect on tetanus or diphtheria immunization status	Decreased total B cells	(17, 111–118)
Rozanolixizumab	36	Decreased IgG (no effect on IgA, IgD, IgE or IgM)	No change in infection incidence	No effect on tetanus or influenza immunization status	No effect on B cells	(7)
Thiopurines	102	Decreased IgA, IgG, and IgM	No change in infection incidence	No effect on pneumococcal, tetanus or *haemophilus influenzae* type B vaccine immunization status	–	(119–122)

Drugs given as chemotherapy include alkylating agents (cyclophosphamide, chlorambucil, bendamustine), corticosteroids, and purine analogs (fludarabine, cladribine, and thiopurines) (6). Treatment with alkylating agents is known to be associated with the development of myelosuppression, during which common infections include pneumonia and bacteremia, caused predominantly by *S. aureus, Streptococcus pneumoniae, H. influenzae*, and *Klebsiella pneumoniae* (6). Purine analogs and purine synthesis inhibitors (such as mycophenolate mofetil) inhibit DNA synthesis, thereby reducing T and B cell proliferation. Use of these therapies, therefore, is more commonly associated with opportunistic infections (e.g., VZV, *Listeria monocytogenes*, and *Candida* spp.) in patients with hematological malignancies (6).

It is well-known that high-dose and long-term treatment with systemic steroids exerts immunosuppressive effects on cellular immunity; however, there is a growing appreciation of the impact on antibody production. A study of the prevalence of hypogammaglobulinemia in 36 patients with giant cell arteritis and polymyalgia rheumatica on glucocorticoid therapy reported that approximately half of the patients developed IgG deficiency with less impact on IgA and IgM and a reduction in naïve B cells with relative preservation of class switched memory B cells (73).

Importantly, diagnostic findings such as this relatively IgG-specific effect of glucocorticoid therapy, can be used clinically to help determine the etiology of antibody deficiency (primary or secondary), a distinction which is diagnostically challenging (73). It is particularly difficult to determine causality of antibody deficiency following administration of therapies known to potentially cause secondary antibody deficiency, especially in situations where both the disease and the treatment used can cause secondary antibody deficiency (e.g., CLL), as well as when multiple lines of immunosuppressive therapy have been used over time (5, 73). In the case of heart transplantation, single center, multi-center, and metanalysis studies have demonstrated the role of immunological monitoring of humoral immunity using similar tools and cut-offs as those used in the diagnosis of PAD (124–126).

There are also diseases other than hematological malignancies that are associated with an increased risk of secondary antibody deficiency following treatment. For example, in antineutrophil cytoplasmic antibody-associated vasculitis (AAV), secondary antibody deficiency of IgG <4 g/L occurs commonly, presenting in 9% of patients after rituximab induction and in a further 4.6% of patients with maintenance therapy in those with low initial IgG levels (127). This degree of secondary antibody deficiency is higher than observed for the long-term combination of rituximab and methotrexate in rheumatoid arthritis, where reduced levels of IgG, IgA, and IgM were observed in 3.5, 1.1, and 22.4% of patients, respectively (128). It is of course difficult to determining whether this increase in the incidence of secondary antibody deficiency is clearly disease specific, but the findings do suggest that a high index of suspicion is needed in this condition in particular.

In neurology, B cell ablation is being increasingly used, for conditions including neuromyelitis optica spectrum disorders, relapsing-remitting multiple sclerosis, myasthenia gravis, autoimmune encephalitis, immune mediated peripheral neuropathies and primary angiitis of the central nervous system, with reports of secondary antibody deficiency consequent upon the use of these agents (129, 130). Clozapine, a drug used in treatment-resistant schizophrenia, has been associated with an increased incidence of pneumonia and death from infection. It has also recently been found to be associated with secondary antibody deficiency, as demonstrated by levels of IgG, IgA, and IgM below the reference range in 8.5%, 13.8%, and 34% of patients, respectively (70).

### Newer Therapeutic Agents That Can Cause Secondary Antibody Deficiency

While the risk of secondary antibody deficiency in patients with CLL and MM and those treated with traditional chemotherapeutic agents has been relatively well-described in older studies, there have been major therapeutic advances which have significantly influenced the risk of iatrogenic secondary antibody deficiency. Amongst these advances, of particular relevance are the many new B cell targets shown in Figure 2 (2, 6). These treatments either deplete B cells (anti-CD20, anti-CD52, anti-CD74, anti CD19, anti-CD22) and plasma cells (anti-CD38) or inhibit B cell survival (anti-B-cell activating factor [BAFF]), impair activation (proteasome inhibitors, tyrosine kinase inhibitors) or interaction with T cells (anti-CD80/86) (2) and all have the potential to cause iatrogenic secondary antibody deficiency. Bruton's tyrosine kinase (BTK) inhibitors, such as ibrutinib, target BTK to inhibit B cell survival and proliferation (Figure 2), and are increasingly used in CLL (16, 131, 132), with severe infections of Grade 3 or higher reported in up to 35% of patients (132). Blinatumomab, a bispecific T cell engager (BiTE) antibody, crosslinks CD3 on T cells to CD19 on B cells activating endogenous T cells to proliferate and become cytotoxic to CD19-positive B cell targets (133) and, as such, is also expected to have the potential to cause secondary antibody deficiency. The phosphoinositide 3-kinase δ inhibitor idelalisib, approved for the treatment of CLL, follicular lymphoma, and small lymphocytic lymphoma, has been associated with increased risk of infections, including pneumonia (92). In addition to these, there is a rapidly increasing number of other new anti-B cell agents, including those targeting molecules expressed by B cells throughout different parts of their developmental pathway such as CD19, CD20 CD22, CD38, CD74, and proteasomes (Figure 2) (38, 96, 134–148).

Given the relatively recent development of some of these therapies, evidence for secondary antibody deficiency during their use is limited by study size, disease setting, and duration of therapy.

In the case of anti-CD19 agents, reductions in all immunoglobulin classes have been reported (although still falling within the reference range) during a small study of inebilizumab in multiple sclerosis (96) and hypogammaglobulinemia was reported in 6% of patients treated with blinatumomab (149). There is more limited information on the incidence of secondary antibody deficiency for the anti-CD20 antibodies ofatumumab and ocrelizumab, however an increase in infection incidence has reported for ocrelizumab (150).

Results thus far have shown a reduction in IgM but not IgG for the anti-CD22 antibody epratuzumab (78), and a potential increase in the incidence of VZV infection with the anti-CD38 agent daratumumab which targets plasma cells (151). The proteasome inhibitor bortezomib has been associated with increased incidence of VZV infection, in addition to herpes simplex virus infection (29). For the anti-CD74 agent milatuzumab, currently in development, there are as yet no published data related to secondary antibody deficiency or risk of infection, and for all of these more recently-developed agents, longer-term studies will be needed to define the degree of impact on immunoglobulins in different disease settings.

Interestingly, anti-BAFF therapy with belimumab has not been associated with a reduction in IgG, pneumococcal antibodies or an increase in infection, and IgM memory B cells have been shown to be reduced while class switched memory B cells were preserved (38, 39). For the transmembrane activator and calcium-modulating cyclophilin ligand interactor-Ig fusion protein atacicept, used in systemic lupus erythematous and rheumatoid arthritis, a reduction in all immunoglobulins has been reported, although this was not linked to infection (152–154).

A good example of increased potency of some of the new therapies are chimeric antigen receptor (CAR)-T cells with synthetic, engineered receptors targeting B cells (e.g., anti-CD19) which are used for the treatment of hematological malignancies (155–157). These engineered T cells can proliferate and retain effector functions of the activated T cells. The likelihood of iatrogenic secondary antibody deficiency with CAR-T therapy is so high that B cell depletion is acknowledged as an “expected on-target result” which may require the use of IgRT (155).

### Future Therapeutic Agents That Could Cause Iatrogenic Secondary Antibody Deficiency

Novel therapeutic strategies continue to be identified. The tetravalent bispecific anti-CD19/CD3 T and Ab tandem diabody AFM11, which consists only of Fv domains, with two binding sites for CD19 on B cells and two for CD3 on T cells, was designed to exhibit higher potency than blinatumomab owing to the bivalent binding of the Fv domains to both B and T cells, resulting in enhanced B cell lysis (158). These immune cell-recruiting bispecific antibodies harness the cytotoxic potency of endogenous T cells (or in some cases NK cells) to kill both malignant and normal B cells (158).

Yet other treatments are becoming available that may be able to treat their target diseases not by interfering with antibody production but by removing pathogenic autoantibodies by reduction of their half-life. Rozanolixizumab, for example, is an anti- FcRn monoclonal antibody (Figure 2) (7). FcRn salvages and recycles plasma IgG autoantibodies, thus extending their lifespan (7, 159). Selective inhibition of FcRn, and hence the salvage pathway, offers a new approach for the removal of pathogenic IgG autoantibodies in the treatment of autoantibody-mediated disease. While pre-clinical and clinical studies of rozanolixizumab have not shown an increase in the incidence of infection (7), long-term studies and close monitoring will be needed. As mentioned above, the risk of infection appears lower where there is removal (plasma exchange, anti-FcRn mAb) or loss (protein-losing enteropathy or renal loss) of functionally normal antibody rather than a production failure. This may translate into a lower infection risk with anti-FcRn therapies; however, long-term data will be required and it would seem prudent to optimize protection from infection by ensuring appropriate vaccination prior to commencement of therapy.

### Solid Organ Transplantation (SOT)

**Figure 3:**
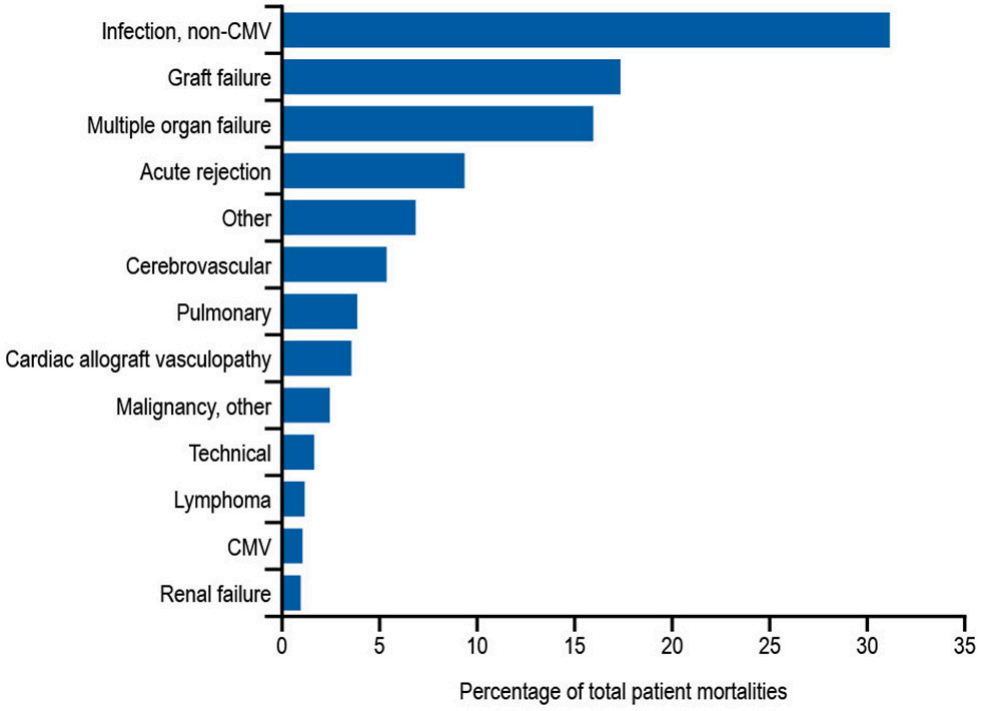
Relative incidence of leading causes of death (31 days to 1 year) in adults receiving heart transplants Jan 1994–June 2016. Developed using data from The International Society for Heart & Lung Transplantation, (2017) (163); CMV, cytomegalovirus.

In addition to the risks associated with hematological malignancy and immunosuppressive drugs, there are also other, perhaps less well-recognized situations in which patients are at risk of developing secondary antibody deficiency. Solid organ transplantations (SOTs) are now routine surgical procedures for treating organ dysfunction, with an estimated 119,873 SOTs performed worldwide in 2014, and 30,970 in the US alone in 2015 (160). Patients receiving SOT are given immunosuppressive drugs before, during, and after transplantation to prevent organ rejection (161). While immunosuppressive drugs are recognized as a common cause of secondary antibody deficiency, there are additional risks in SOT including the transplantation procedures themselves as well as other interventions, such as the use of ventricular assist devices in heart recipients or extracorporeal membrane oxygenation in heart and lung recipients, which are also associated with the occurrence of SOT-related secondary antibody deficiency (162, 163). Several studies have shown a high prevalence of hypogammaglobulinemia after SOT, particularly after heart, lung, and kidney transplantation, with an associated increased risk for infections (124, 164). Infections, most frequently non-CMV infections, are reported to be the leading cause of death during the first year after heart or lung transplantation (Figure 3) (163). In one meta-analysis of 669 SOT patients, 15% developed severe hypogammaglobulinemia (IgG < 4 g/L) during their first year post transplantation (124). This study also reported a 4.8-fold increase in respiratory infections, a 2.4-fold increase in CMV infections, and an 8-fold increase in aspergillus infections (3.7-fold increase in other fungal infections) when severe hypogammaglobulinemia was present (124). Furthermore, two other groups have also reported an increased incidence of opportunistic infections in heart transplantation with hypogammaglobulinemia, particularly CMV viremia (126, 162). In addition, lower levels of anti-pneumococcal antibodies are a risk factor for development of severe bacterial infections in heart recipients (165).

## Diagnosis

The prompt diagnosis of secondary antibody deficiency is key in reducing infection burden and is dependent on appropriate screening and an appreciation of risk factors for secondary antibody deficiency development. Monitoring of patients at risk of developing secondary antibody deficiency, such as patients receiving conventional immunosuppressive drugs or newer, more targeted therapies, could help in identifying these patients before they develop a serious infection. For rituximab, which is known to be associated with the development of secondary antibody deficiency, the risks for an individual are increased with low baseline IgG prior to rituximab initiation; exposure to prior therapies, such as cyclophosphamide, steroids, and purine analogs, purine synthesis inhibitors (mycophenolate mofetil), or the use of combination therapy or granulocyte-colony stimulating factor (166). Furthermore, the single use of rituximab rarely causes hypogammaglobulinemia, yet there is an increased risk for patients undergoing maintenance or multiple cycles of treatment. Finally, patients with co-morbidities, such as chronic lung or heart disease and extra-articular rheumatoid arthritis, have higher instances of hypogammaglobulinemia, with more infections occurring when IgG is low for longer than 6 months (166). Although there may also be particular non-hematological diseases which have higher rates of secondary antibody deficiency, such as AAV and neuromyelitis optica spectrum disease, the caveat remains that it is difficult to separate the effect of treatment from disease.

The evaluation of patients at high risk or with suspected hypogammaglobulinemia should include quantitative serum Ig concentrations and analysis of vaccination responses if initial specific antibody levels are found to be low. High-risk patients may merit annual immunoglobulin measurements to screen for the development of secondary antibody deficiency. It is likely that in some cases it will be helpful to determine levels and reconstitution of B cell numbers, including the preservation of class-switched memory B cell and plasma cell compartments. For example, in some settings the measurement of the return of class-switched memory B cells is already being used to determine the timing of retreatment with rituximab to reduce the cumulative rituximab dose without apparent loss of efficacy (167). In addition, complete blood count will assist in identifying additional risk factors such as neutropenia, lymphopenia, or lymphocytosis (2, 168).

Some treatment-specific screening or monitoring programs have been introduced to mitigate infection-related risk; for example, the Pharmacovigilance Risk Assessment Committee of the EMA recommends that patients receiving idelalisib should be informed about the risk of serious and/or fatal infections, given prophylaxis for *Pneumocystis jirovecii* pneumonia, and monitored for signs and symptoms of respiratory infections throughout the idelalisib treatment. Regular clinical and laboratory screening for CMV infection should also be conducted and absolute neutrophil counts monitored in all patients at least every 2 weeks for the first 6 months of treatment. In the case of clozapine, the Clozapine Risk Evaluation and Mitigation Strategy (REMS) monitoring scheme has been established to reduce the risks associated with neutropenia, however antibody monitoring is not currently part of the scheme (169).

**Figure 4:**
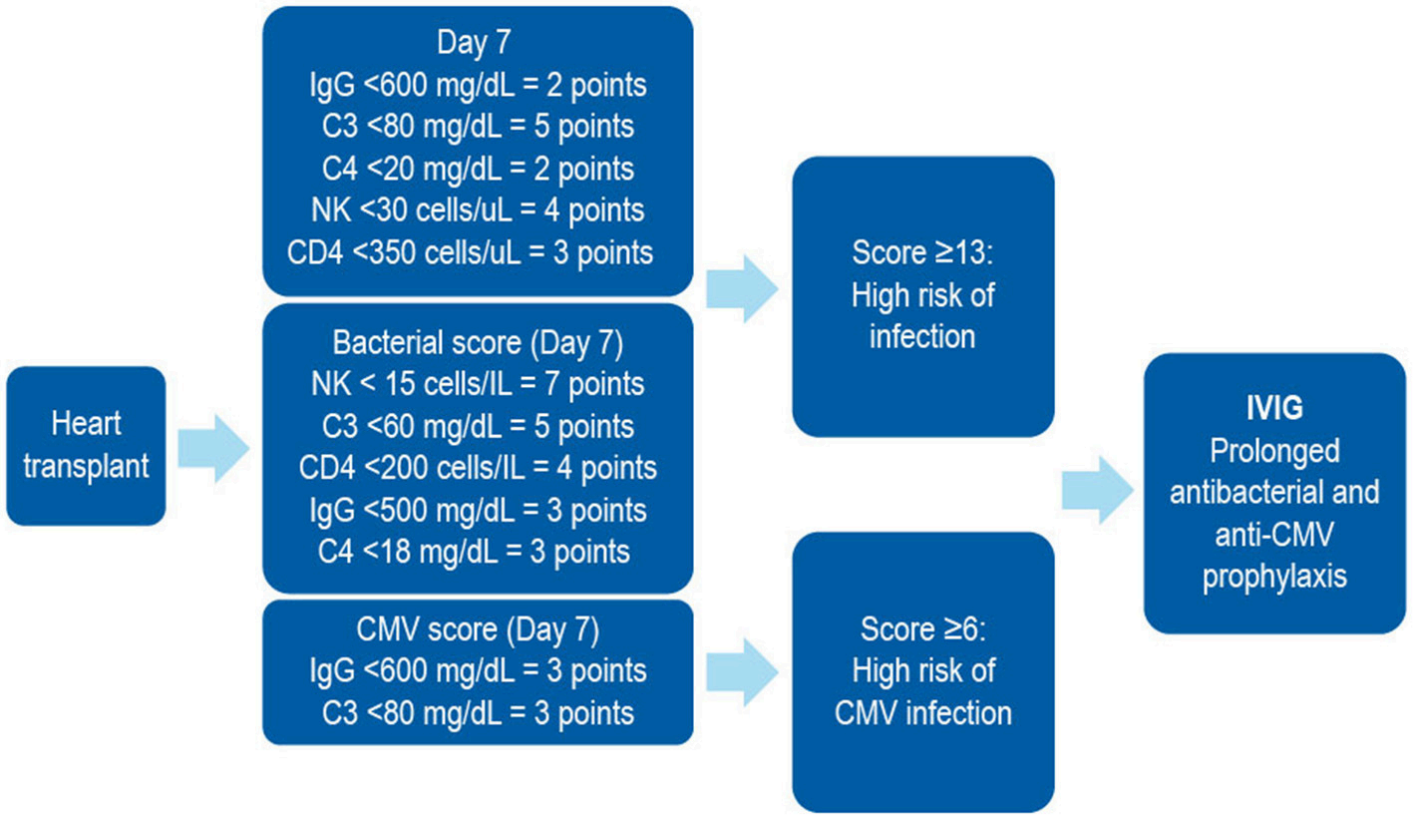
A combined immunodeficiency profile identifies risk of severe infection in heart transplant recipients. Developed using data from Sarmiento et al. (186). C, complement; CD, cluster of differentiation; CMV, cytomegalovirus; IVIG, intravenous immunoglobulin; NK, natural killer.

In patients receiving SOT, several biomarkers have been shown to be useful in stratifying the risk of developing SOT-related secondary antibody deficiency and associated infections. For example, in patients receiving a heart transplantation, IgG levels of < 10 g/L prior to transplantation may predict high frequency of bacterial infections, therefore suggesting that monitoring of IgG levels prior to transplantation may be important in predicting secondary antibody deficiency and subsequent infection. Similarly, low levels of class-switched memory B cells may also act as pre-heart transplantation biomarkers. Several post-heart transplantation biomarkers, such as low peripheral lymphocyte populations, may also help in predicting infections. For example, low anti-CD8 response to CMV antigens assessed by enzyme-linked immune absorbent spot (ELISPOT) or flow cytometry can be used as a biomarker for the risk of developing CMV infection and disease (170). Other prospective post-SOT biomarkers include complement components (e.g., C3 and C4), owing to their effector functions in the innate and adaptive humoral immune responses (171). The classic *in vitro* hemolytic assays utilized for assessing classical and alternative complement pathway function are complex, susceptible to pre-analytical sample handling issues, and time consuming. Nephelometry represents a convenient option for the measurement of certain complement components in serum (172). As complement activation is responsible for the clearance of encapsulated bacteria, hypocomplementemia may result in increased susceptibility to infection (171). It has been shown that hypocomplementemia is an indicator of bacterial infection risk in patients following kidney transplantation (173), with similar findings reported for liver (174) and heart (175) recipients. The combination of low IgG and low C3 in serum can be particularly detrimental in SOT patients. One multivariate analysis demonstrated that hypogammaglobulinemia (IgG <6 g/L) or C3 hypocomplementemia (C3 <80 mg/dL) on Day 7 post heart transplantation were independent risk factors for infection (especially bacterial infections) and CMV disease (125). However, the combination of both was more strongly associated with the risk of infections than either hypogammaglobulinemia or C3 hypocomplementemia alone. Furthermore, on Day 7, low anti-CMV antibody titers and low anti-pneumococcal polysaccharide antibody concentrations as biomarkers of impaired specific antibody responses were independent predictors of CMV disease and bacterial infections, respectively (125). These biomarkers can be used to identify patients at high risk who may benefit from IgRT post heart transplantation (Table 2, Figure 4). The evaluation of CMV IgG serology of donor and recipient is a classic evaluation to identify the risk of CMV disease after SOT (170). CMV seronegative recipients receiving an allograft from a CMV seropositive donor are considered to be at higher risk of having this complication than other serological combinations. More recently, the assessment of CD8 responses to CMV antigens has also been introduced to evaluate the risk of CMV disease in SOT (170).

**Table 2:** Biomarkers used following heart transplantation.

**Organ**	**Biomarker (post HT)**	**Outcome**	**References**
Heart	IgG <3.5 g/L	Opportunistic infection	(162)
Heart	IgG <3.1 g/L	Opportunistic infection	(176)
Heart	IgG <6 g/L	Bacterial, CMV infection	(126)
Heart	IgG <7 g/L	Bacterial, CMV infection	(165)
Heart	IgG <4 g/L	Clostridium difficile	(177)
Heart	IgG <5 g/L	CMV disease	(178)
Heart	Low IgG2	Overall infection	(175)
Heart	anti-PPS <5 mg/dL	Bacterial infection	(165)
Paed heart	Low anti-PPS	Not done	(179, 180)
Heart	Low anti-CMV titres	CMV infection	(178, 181, 182)
Heart	C3 <80 mg/dL	Overall infection	(183) First proposal of an immunological score
Heart	NK <30 cells/uL		
Heart	CD4 <350 cells/uL		
Heart	Low anti-CD8 response to CMV	CMV infection	(183–185)

*C3, complement component 3; CD, cluster of differentiation; CMV, cytomegalovirus; HT, heart transplant; IgG, immunoglobulin; NK, natural killer; PPS, pneumococcal polysaccharide*.

An interesting aspect of the management of heart and other SOT recipients is that vaccination is not common practice during the first months after transplantation, when the majority of severe infections occur, because the specific antibody response after immunization in this period has been demonstrated to be low (187). This should be taken into account at the time of diagnosing secondary antibody deficiency in this setting. Moreover, the titers of anti-pneumococcal or anti-CMV antibodies gradually decline during the first year after transplantation as a result of the immunosuppressive therapy, leaving the patients exposed to infection (165, 178, 188).

## Treatment

While removal of the underlying causative factor would be the preferred option for treating secondary antibody deficiency, (e.g., as a consequence of the removal of an abnormal section of bowel in protein-losing enteropathies or reduction of immunosuppression post hematopoietic stem cell transplantation [HSCT]) this is overall rarely possible, particularly when treatment cannot be easily avoided as is the case with SOT, CLL, MM, or lymphoma. Indeed, in the case of HSCT, this may also itself lead to secondary antibody deficiency and subsequent infection, the risks of which are increased by the presence of graft vs. host disease (GvHD) in these patients (189, 190). While the mechanism of GvHD-mediated secondary antibody deficiency is not fully understood, it is thought to potentially delay B cell engraftment, leading to B cell lymphopenia, and to delay/prevent B cell maturation and memory development, markedly decreasing memory and switched memory B cells (191, 192).

In hematological malignancy, supportive treatments including prophylactic vaccination (with non-live vaccines), antibiotics and/or IgRT may be considered for patients with secondary antibody deficiency. A careful review of the patient's history, assessment of the risk factors for developing secondary antibody deficiency and evaluation of serum IgG levels and specific antibody levels is key to the diagnosis and treatment. Immunologists often have greater experience in managing PAD than secondary antibody deficiency, and it is likely that the clinical and laboratory assessments used in this setting would also be of utility in secondary antibody deficiency. However, there is limited evidence in the literature, particularly relating to the increasing number of newer therapies, to guide clinical management of secondary antibody deficiency.

### Prophylactic Vaccination

Prophylactic non-live vaccinations such as influenza are recommended for patients with secondary antibody deficiency, as is the case in PAD. Although the antibody response may not be optimal, some helpful protection via antibodies and T cell immunity may still be achieved; thus, influenza vaccination is advised. Live vaccination is generally not recommended in patients with secondary antibody deficiency (although individual assessment and decisions are warranted). Instead, component or inactivated vaccines should be used. Studies suggest that vaccination at an early stage (before initiation of chemotherapy and the onset of hypogammaglobulinemia) may be more helpful in generating immunological memory when specific antibody levels are low (123). In addition, evaluation of post-vaccination–specific antibody levels can be beneficial both therapeutically (if protective levels are achieved) and in stratification of risk before and following treatment. Vaccination against *S. pneumoniae* and *H. influenza* is recommended in patients with CLL; however, the protective effect of these vaccines may be variable, emphasizing the importance of ongoing immunological assessment and individualization of care (6). In a UK-based study of patients with MM, 61% (26/43) showed a suboptimal response to *S. pneumoniae* vaccination, whereas 75% (33/46) showed a response to *H. influenzae* type B vaccination comparable with those in the healthy adult UK population. The European Myeloma Network guidelines (193) suggest that “*vaccination against influenza virus is appropriate and is recommended for both patients and their contacts. Moreover, vaccination against Streptococcus pneumoniae and Haemophilus influenzae is recommended, but efficacy for all vaccines is not guaranteed, due to suboptimal immune response (grade 1C). In general, live vaccines should be avoided in myeloma patients (grade 2C)”* (193).

The Infectious Diseases Society of America has recommended the routine use of inactivated vaccines in patients with MM unless they are actively receiving chemotherapy or monoclonal antibody (194). The Advisory Committee for Immunization Practices recommends that patients with MM and SOT should receive vaccination with pneumococcal conjugate vaccine at diagnosis followed by revaccination more than 8 weeks later (195). Recent MM guidelines from the National Comprehensive Cancer Network do not include specific recommendations for vaccination, but do note to “*consider Pneumovax and influenza vaccine*” (196).

In terms of pre-transplantation vaccination schedules, it is suggested that primary immunizations should be given as soon as possible before transplantation (197). Data from a study of pediatric patients showed that vaccination prior to transplantation was effective in reducing the incidence of *Varicella Zoster* virus infection from 45–12% (198). Guidelines for vaccination of SOT recipients recommend vaccination against *S. pneumoniae* and *H. influenzae* before transplantation (197, 199). As mentioned, post-transplantation, anti-pneumococcal antibody titers have been shown to significantly decrease in kidney (188) and lung transplant recipients (200). Questions remain regarding the utility and timing of vaccination in secondary antibody deficiency and further studies are needed (6).

While it is generally accepted that inactivated vaccines can be administered in patients with secondary antibody deficiency, many vaccinations are not required for those patients receiving IgRT, as protective levels of antibodies are already present in the IgRT preparations and should offer some protection against a range of infectious diseases (201, 202). Influenza vaccination is an exception, as these vaccines are reformulated annually to reflect changes in the antigenic composition of the influenza virus and protection from IgRT is therefore less likely. However, even if the antibody response post vaccination is suboptimal, patients with PAD and also secondary antibody deficiency may benefit from the T cell-mediated responses (112, 203). Live vaccines are generally not recommended in the context of significant immunodeficiency and in patients receiving IgRT may also be less effective (204).

### Prophylactic Antibiotics

Treatment of secondary antibody deficiency with prophylactic antibiotics is the recommended first-line therapy in CLL (205) and during periods of neutropenia in patients undergoing chemotherapy or other immunocompromising treatments (11). Antibiotic therapy should take into account the previous culture and sensitivity results as well as any allergies, tolerance, and the likelihood of *Pseudomonas* or macrolide-resistant *H. influenzae* infection. In the event of a breakthrough infection, if there has been no or limited response to a back-up course of antibiotics and a second course of different antibiotics, then intravenous antibiotic (IVAB) treatment should be considered. Prophylactic and back-up antibiotics should be of different classes (e.g., macrolide and penicillin class), rather than simply increasing the dose of the existing prophylactic regimen. Monitoring (e.g., electrocardiogram) and additional patient information (e.g., regarding the development of tinnitus) may be needed for those on long-term macrolides. There are many potential antibiotic options and the examples shown in Table 3 are illustrative, with individual decisions being made on clinical grounds and local prescribing policy. Nebulized antibiotics and intermittent IVAB are options that are used mainly for severe bronchiectasis and pseudomonal colonization (Table 3).

**Table 3:** Graded antibiotic regimens^*^.

**Antibiotic regimen**	**Dosing schedule**	**Additional options**	**Emergency plan**	**Example**
Intermittent antibiotics	None		Attend GP with symptoms	N/A
	None		Early use of home back-up antibiotics	Co-amoxyclav 625 mg tds for 2 weeks; held at home
	Prophylactic antibiotics during the winter months with home rescue during the summer	Low-dose and full-dose options, e.g., azithromycin 250 or 500 mg 3 days/week	Early use of home back-up antibiotics	Azithromycin 500 mg 3 days/week plus back-up Co-amoxyclav for 2 weeks; held at home
Ongoing prophylaxis	Prophylactic antibiotics	Low-dose and full-dose options, e.g., azithromycin 250 or 500 mg 3 days/week	Early use of home back-up antibiotics	Azithromycin 500 mg 3 days/week plus back-up Co-amoxyclav 625 mg tds for 2 weeks; held at home
	Rotating prophylactic antibiotics		Early use of home back-up antibiotics	
	Prophylactic antibiotics	Nebulized antibiotics	Early use of home back-up antibiotics	Nebulized Colomycin 1–2 mega units bd
	Prophylactic antibiotics	Intermittent planned IVAB	Early use of home back-up antibiotics	Meropenem 2g IV tds and Ceftazidime Co-amoxyclav for 2 weeks; held at home

*GP, general practitioner; IV, intravenous; IVAB, intravenous antibiotic; N/A, not available; bd, twice daily; tds, three times daily*.

* *If there has been an inadequate response to back-up antibiotics and an additional antibiotic in another class then intravenous antibiotics (IVAB) should be considered. The Table shows examples of antibiotic regimens and the antibiotic choice will depend on individual clinical circumstances*.

### Current Use of IgG

Many studies have demonstrated decreased infection incidence in patients with CLL treated with intravenous IgG (IVIG; Table 4). The beneficial effect of IVIG was demonstrated in a randomized, controlled, double-blind clinical trial conducted by the Cooperative Group for the Study of Immunoglobulin, where use of IVIG was associated with lower incidence of bacterial infections and longer time during which patients were free of serious bacterial infections compared with patients not receiving IVIG (206). A subsequent double-blind crossover follow-up study confirmed that maintenance on IgG results in reduction of the infection incidence (210). Later, another double-blind crossover study compared two doses of IVIG (250 and 500 mg/kg) over a year, suggesting a significant difference in the incidence of bacterial infections between doses (208). These promising results prompted a consensus statement supporting the use of IVIG in patients with CLL with hypogammaglobulinemia and associated infections (213). However, as these studies are well over a decade old, there is a clear need for newer randomized trials in this area, particularly given the vast advances in treatment and improved survival.

**Table 4:** Summary of evidence for the use of immunoglobulin replacement therapy in CLL.

**Reference**	**Number of patients**	Number of patients in advanced stage^*^	**Type of study**	**Dose IVIG/schedule**	**Study duration (months)**	**Infection rate during IVIG administration**
Cooperative group (206)	81	32 (39.5%)	Controlled, randomized double-blind	400 mg/kg/ 21 days	12	Decreased
Jurlander et al. (207)	15	8 (53.3%)	Not controlled, pilot	10 g/28 days	12 (mean time)	Decreased
Chapel et al. (208)	34	15 (44.1%)	Controlled, randomized double-blind	250 mg/kg vs. 500 mg/kg/28 days	12	Decreased
Sklenar et al. (209)	31	2 (6.4%)	Dose-finding	100–800 mg/kg/ 21 days	4.5	Decreased
Griffiths et al. (210)	10	3 (30%)	Controlled, randomized double-blind	400 mg/kg 21 days	12	Decreased
Broughton et al. (211)	42	15 (35.7%)	Randomized	18 g/21 days	12	Decreased
Molica et al. (212)	30	25 (83.3%)	Randomized, crossover	300 mg/kg/28 days	6 or 12	Decreased

* *Binet stage C or Rai III-IV*.

Protocols for IgRT in secondary antibody deficiency vary. In clinics based in Oxford and Cardiff, UK, very similar approaches are employed where the use of IgRT and the dose administered are individualized per patient (5). In patients referred to the immunology department for recurrent and/or serious bacterial infections, immunization responses are measured 4–6 weeks after administration of protein and polysaccharide vaccines (if initial levels are low) and during the waiting period, for up to 3 months, prophylactic antibiotic use is considered. A 12-month trial of IgRT (with infection monitoring) is considered in case of antibody failure and a lack of an adequate response to prophylactic antibiotics in association with a significant ongoing infection burden. Neutrophil count, serum IgG trough levels, and infection burden should be monitored regularly, and IgG use, including dosage, should be reviewed after 6 and 12 months to maintain plasma IgG trough levels sufficient for an optimal reduction in infection rate. Levels of IgG should be reviewed if chemotherapy is introduced during IgRT. Because patients react differently to IgRT, individualization of the treatment regimen is required (5).

**Figure 5:**
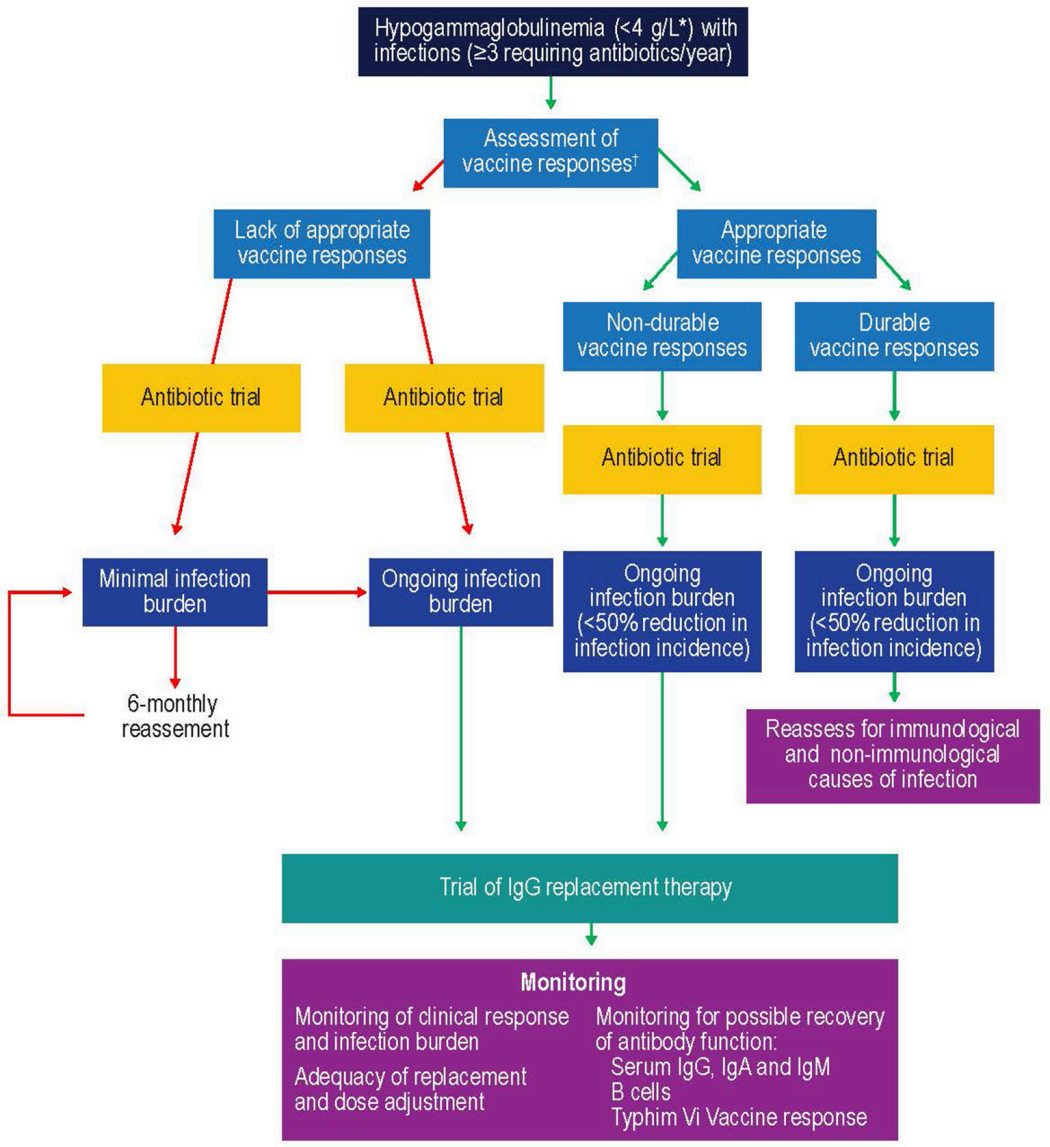
Suggested protocol for the investigation, monitoring, and management of secondary antibody deficiency. Reproduced with the permission of the copyright holder John Wiley & Sons Inc (5). ^*^See (57, 58, 214); ^†^Only >6 month after solid organ transplant. CSMB, class-switched memory B cells; IgA, immunoglobulin A; IgG, immunoglobulin G; IgM, immunoglobulin M; SOT, solid organ transplant.

One of the key factors to consider when initiating IgRT is selection of patients most likely to benefit (Figure 5). Studies suggest that patients with IgG levels <4 g/L and/or low levels of antibodies against encapsulated organisms with an ongoing history of recurrent bacterial infections that have not responded adequately to prophylactic antibiotics could especially benefit from IVIG (211, 215). Furthermore, selection of the patients should include a wider assessment of comorbidities and innate immunological abnormalities, such as neutropenia, as well as demonstrating antibody failure (exposure/test immunization). If IgRT is to be administered, a careful risk-benefit assessment should be made. This should take into consideration, for example, the reported adverse effects of IgRT, such as the rare complications of thromboembolism and hemolysis in patients with hematological malignancies (216, 217). Once it is established that IgRT should be offered to a patient with secondary antibody deficiency, the route [subcutaneous IgG [SCIG] or IVIG] and location (home vs. clinic) of infusions should be considered. SCIG has similar efficacy to IVIG (218, 219) but offers several potential benefits, including more stable serum IgG levels (219, 220), improved patient health-related quality of life (221), and time and cost efficiencies for both patients and healthcare providers. It has also previously been shown that the feasibility and safety of home IVIG therapy in selected CLL patients may improve patient compliance and considerably decrease costs (222). It is also recommended that patients complete at least 12 months (encompassing all 4 seasons) of IgRT in order to optimally assess the response to therapy (11). This will require ongoing review to reflect any clinical changes in the patient's underlying condition and therapy. Treatment discontinuation may be considered in stable patients with reduced incidence of infections (i.e., once treatment has restored function) to determine if IgRT is still required, or in patients where treatment does not seem to be effective in preventing infection (i.e., treatment failure) (11).

Prophylactic use of IgRT in patients post SOT has been shown to decrease the incidence of severe infection in patients with hypogammaglobulinemia (162, 223). It has also been shown to decrease the incidence of CMV infection in patients with hypogammaglobulinemia (224). Retrospective studies have demonstrated that addition of IVIG to antimicrobial therapy in heart transplantation recipients with secondary antibody deficiency and severe infections is associated with reconstitution of specific antibodies (anti-CMV, anti-tetanus toxoid) and lower rates of death (183, 225). A case series performed in heart transplantation recipients with difficult-to-control CMV infection in whom secondary antibody deficiency was found demonstrated that IVIG was associated with reduction in detectable CMV viremia and with remission of clinical symptoms of CMV disease (226).

## Current Evidence-Based Guidelines for IgRT in Secondary Antibody Deficiency

The majority of guidelines recommend prophylactic antibiotics as first-line therapy for patients with CLL susceptible to serious infections (2). However, an individualized dose and duration of IgRT is also suggested where there is lack of responsiveness or failure of the antibiotics to sufficiently reduce the infection burden in such patients. Looking at specific recommendations, according to European Medicines Agency (EMA) 2018 (valid from January 2019); IVIG can be used in patients with secondary immunodeficiencies “*who suffer from severe or recurrent infections, ineffective antimicrobial treatment and either proven specific antibody failure (PSAF) or serum IgG level of* <*4 g/l,” where* PSAF is defined as “*failure to mount at least a 2-fold rise in IgG antibody titer to pneumococcal polysaccharide and polypeptide antigen vaccines*” *(214)*. The dose required is stated to be probably patient dependent, but is likely to be 0.2–0.4 g/kg every 3–4 weeks (214). With regard to SCIG, EMA guidelines state that it is indicated in hypogammaglobulinemia and recurrent bacterial infections in patients with MM and in patients with CLL in whom prophylactic antibiotics have failed or are contraindicated (227). SCIG is also indicated for hypogammaglobulinemia in patients pre- and post-allogeneic HSCT. However, the EMA states that “*the above indications would be granted as long as efficacy has been proven in primary immunodeficiency syndromes*” (227).

The European Society for Medical Oncology (ESMO) guidelines, published in 2015 (228), state that “*the use of prophylactic systemic IgG [*in patients with CLL*] does not have an impact on overall survival and is only recommended in patients with severe hypogammaglobulinemia and repeated infections [Level of evidence I, A]*.” They also state that “*Antibiotic and antiviral prophylaxis should be used in patients with recurrent infections and/or very high risk of developing infections (e.g., pneumocystis prophylaxis with co-trimoxazole during treatment with chemoimmunotherapies based on purine analogs or bendamustine) [IV, B]*” and finally, that “*pneumococcal vaccination as well as seasonal influenza vaccination is recommended in early-stage CLL [IV, B]*” (228).

According to The British Committee for Standards in Hematology, 2012 (205), ” *in the absence of recent randomized studies, recommendations for the use of immunoglobulin replacement in CLL are largely based on clinical experience and data from use in primary immunodeficiencies*.” Their recommended indication for the use of IgRT is “*recurrent/severe infection with encapsulated bacteria despite prophylactic oral antibiotic therapy in patients with a serum IgG* <*5 g/L (excluding paraprotein)*” (205).

Guidelines published in 2011 by the UK Department of Health (229) recommend that “*Immunoglobulin replacement therapy is recommended in secondary antibody deficiency if the underlying cause of hypogammaglobulinaemia cannot be reversed or reversal is contraindicated, or is associated with B-cell malignancy where severe infections with encapsulated bacteria are persistent despite prophylactic antibiotic therapy (grade C recommendation, level III evidence)”* (229).

The Criteria for Clinical Use of Immunoglobulin from the National Blood Authority in Australia provide a variety of recommendations for IgRT, including: “*Prevention of recurrent bacterial infections due to hypogammaglobulinaemia associated with hematological malignancies or post haemopoietic stem cell transplant” (230)*, and “*replacement therapy for recurrent or severe bacterial infections or disseminated enterovirus infection associated with hypogammaglobulinaemia caused by a recognized disease process or B cell depletion therapy and/or immunosuppressant therapy*” (231). Finally, in Canada, guidelines published in 2018 state that “*Immunoglobulin replacement is recommended for preventing recurrent, severe infection due to hypogammaglobulinemia (excluding paraprotein) related to other diseases or medical therapy” (232)*. Specifically, secondary antibody deficiency is defined as “*Hypogammaglobulinemia secondary to underlying disease or medical therapy (including HSCT) with all of the following: Serum IgG less than the lower limit of the reference range on two separate occasions AND at least one of the following: a) one invasive or life-threatening bacterial infection (e.g., pneumonia, meningitis, sepsis) in the previous year; b) recurrent, severe bacterial infections; c) Clinically active bronchiectasis confirmed by radiology or d) assessment by a physician specializing in immunodeficiency indicating a significant antibody defect that would benefit from immunoglobulin replacement” (232)*.

In the field of SOT, there are currently no published guidelines regarding the use of IgRT in patients with recurrent bacterial infections and hypogammaglobulinemia. In the updated International Consensus Guidelines on the management of CMV in SOT, published by the Transplantation Society International CMV Consensus Group, there is a recommendation that, when CMV disease is difficult to control, IgG testing is advisable (170). According to expert opinion, CMV-specific IgRT is recommended for prophylaxis of CMV disease in high risk CMV seronegative recipients in specific settings, such as thoracic, intestinal, and pediatric transplantation (170). The European Society of Clinical Microbiology and Infectious Diseases guidelines recommend antiviral prophylaxis treatment for patients at high risk of CMV disease, such as lung and intestinal transplant recipients (233).

Overall, most guidelines are in support of considering IgRT initiation in selected patients with secondary antibody deficiency. In patients with risk factors (e.g., cardiovascular disease, diabetes, renal impairment, and thrombosis risk), a careful risk-benefit assessment should determine whether initiating IgRT is advisable. Also, the dose, route, and administration frequency should be optimized for each patient individually to maintain acceptable plasma IgG levels and a substantial reduction of infection rates (234). However, further clinical studies are required to improve and/or validate our current practice in prescribing IgG to patients with secondary antibody deficiency especially given the growth in newer therapies. It is also clear that individual assessment remains paramount, as diagnostic testing remains imperfect and not all patients will fit neatly into guidelines.

## Future Directions

As the number and variety of immunosuppressive therapies increases, so will the incidence of secondary antibody deficiency (5). Accordingly, the EMA are currently in the process of updating their guidelines on the use of IVIG to specifically address secondary antibody deficiency, recommending its use for “*secondary immunodeficiencies in patients who suffer from severe or recurrent bacterial infections, ineffective antibiotic treatment and either proven specific antibody failure (failure to mount at least a 2-fold rise in IgG antibody titer to pneumococcal polysaccharide and polypeptide antigen vaccines) or serum IgG level of* <*4 g/L*” (235). Their recommended dose is 0.2–0.4 g/kg every 3 to 4 weeks. Indeed, there is also increasing evidence that SCIG offers an efficacious and efficient treatment option for secondary antibody deficiency (236).

It should be emphasized that currently, evidence regarding the newer agents is limited by study size, duration, and the number of disease settings, and longer-term prospective follow-up studies will be needed to clearly define the long term cumulative effect on antibody production and thus inform future guidelines.

In PAD, there are screening and treatment guidelines and protocols in place to identify and treat hypogammaglobulinemia (237–239). However, even in these newer, updated guidelines, there remains a paucity of recommendations related to the diagnosis and screening for secondary antibody deficiency or those addressing the treatment of secondary antibody deficiency specifically. As discussed above, vaccine-specific antibody response is a key tool in the diagnosis of secondary antibody deficiency that will benefit from new assays to assess responses to polysaccharide vaccination, such as Typhim Vi (typhoid polysaccharide vaccine, Sanofi Pasteur, UK), which are being increasingly introduced (240, 241). It will also be important to harness pharmacogenetics in patient selection and risk stratification. Future guidelines will need to focus on these under-represented and rapidly growing patient populations to harmonize and optimize screening, diagnostic, monitoring, and management approaches.

### Avoiding the Consequences of Hypogammaglobulinemia

Ongoing advances in testing, earlier screening, reducing diagnostic delay, and optimizing treatment regimens are all ways to reduce the burden of secondary antibody deficiency. Determining the concentration of the serum globulin fraction (calculated globulin), of which immunoglobulins are a major component, is a useful tool for identifying undiagnosed patients with secondary antibody deficiency (242, 243). However, given the variety of causes and settings in which secondary antibody deficiency may present, raising awareness of conditions, and treatments that increase the risk of secondary antibody deficiency is also vital (Figure 5). The identification of biomarkers, such as those following heart transplantation (e.g., low anti-CD8 response to CMV, reduced complement components), will help to stratify patients by risk and improve recognition. In a wider context, clinical biomarkers that may assist in patient identification may include infection burden (particularly sinopulmonary infection), chronic sinusitis, chronic daily sputum production suggestive of bronchiectasis (5), or IgG level (<4 g/L). With regard to infection burden, factors such as infection frequency, duration, severity, and site should be considered, along with rates of antibiotic (particularly intravenous) use and hospitalization. Alongside vaccine responses, it is advisable to consider other factors, such as neutropenia, significant renal impairment, multiple lines of treatments, and ongoing therapy requirement and timing of vaccination (e.g., in relation to HSCT). In addition, performing measurements of potential biomarkers at baseline will enable objective measurement of the outcome of subsequent interventions (5).

## Conclusions

The prevalence and burden of secondary antibody deficiency are substantial and increasing. Secondary antibody deficiency occurs in the majority of patients with hematological malignancies, such as CLL and MM, either as a result of disease-related effects on the immune system or as a side effect of the treatment. Secondary antibody deficiency can also emerge in less well-known at-risk patient populations, such as patients undergoing SOT (due to post-transplantation immunosuppressive therapy) (6), patients with neurological conditions (129, 130) and patients with psychiatric conditions (70). With the growth in new and more effective improved therapies for malignancy and organ transplantation, the incidence of secondary antibody deficiency is likely to increase further, in particular when targeting different and multiple components of B cell development and survival (Figure 2). As the use of these newer, more effective immunosuppressive medications becomes more prevalent, the resultant increased survival in malignancy will be accompanied by a concomitant increase in the prevalence of secondary antibody deficiency. Given the diverse etiologies of secondary antibody deficiency, healthcare practitioners from a wide range of specialties will need to anticipate the possible immunological adverse effects of the new therapeutic agents targeting the immune system. Not surprisingly, secondary antibody deficiency is often missed despite recurrent infections, resulting in delayed diagnosis and intervention (168). Therefore, there is a real need for increased awareness, screening, and improved monitoring of patients at risk of developing secondary antibody deficiency to aid early identification of these patients to avoid infection-related morbidity and mortality.

Improved screening, such as by measuring calculated globulin, is an important goal in the quest for identifying secondary antibody deficiency as early as possible. In addition, improved risk stratification and the use of biomarkers to identify the patients most likely to require interventions, such as IgRT, within each patient population will be key. secondary antibody deficiency patients will be no less individual than PAD patients and should have the same access to treatments once antibody failure has been demonstrated. The limited evidence available in secondary antibody deficiency suggests that there are significant similarities in management approaches with those used for PAD. However, cohort and prospective studies in secondary antibody deficiency are needed to support decisions regarding patient selection and treatment with targeted vaccination, prophylactic antibiotics, or IgRT. These studies should also help to identify any distinctive clinical features that may differ depending on the etiology of secondary antibody deficiency (e.g., drug-induced vs. malignancy vs. specific disease vs. transplantation setting) and how to optimize management of this under-appreciated condition.

## Author Contributions

All authors listed have made a substantial, direct and intellectual contribution to the work, and approved it for publication.

### Conflict of Interest Statement

SJ has participated in advisory boards, trials, projects, and has been a speaker with Baxalta, CSL Behring, Shire, Thermofisher, Swedish Orphan Biovitrum, Biotest, Binding Site, Grifols, BPL, Octapharma, LFB, GSK, Weatherden, Zarodex, Sanofi, and UCB Pharma. SP has been a speaker for CSL and Biotest. JC has participated in advisory boards and projects, and has been a speaker for Grifols, Biotest, Shire, LFB, CSL Behring, Octapharma. He has a grant from the Instituto de Salud Carlos III FIS 1501472 with participation of FEDER funds, a way of making Europe.
